# Health Care Navigators for Perioperative Advance Care Planning

**DOI:** 10.1001/jamanetworkopen.2024.15452

**Published:** 2024-06-18

**Authors:** Alexis Colley, Adrian Valderrama, Victoria Tang, Logan Pierce, Rebecca L. Sudore, Elizabeth C. Wick

**Affiliations:** 1 Department of Surgery, University of California, San Francisco; 2 School of Medicine, University of California, San Francisco; 3 Department of Medicine, Division of Geriatrics, University of California, San Francisco; 4 Department of Medicine, University of California, San Francisco

## Abstract

This cohort study evaluates the addition of a patient-centered intervention to an advance care planning process for older surgical patients.

## Introduction

Advancements in perioperative care have made immediate catastrophic complications of major elective operations rare, yet the need for surgical intervention may signal declining health for older adults. Therefore, the period before a major operation may be an opportune time for advance care planning (ACP).^1^ Barriers to the widespread adoption of ACP in surgery remain.^2^ We evaluated the integration of a patient-centered tool (PREPARE) into an electronic health record (EHR) patient-portal message sent to patients before surgical consultation.^3^ This integration was intended to allow patients to self-engage with ACP while removing dependency on the surgical team for ACP initiation.

## Methods

This retrospective cohort study was conducted at a tertiary academic cancer center using EHR data. The University of California, San Francisco Institutional Review Board deemed this study exempt from review and waived the informed consent requirement because it was not human participant research. We followed the STROBE reporting guideline.

We included patients in 4 surgical clinics (colorectal, hepatobiliary, endocrine, thoracic) who were 65 years or older and scheduled for a new patient visit (June 1, 2022, to June 1, 2023). One week before the visit, patients received an EHR message explaining ACP with a hyperlink to PREPARE (available in English and Spanish). The message was written in English or Spanish, based on the patient’s self-identified preferred language in the EHR. Two multilingual and ACP-trained health care nonclinical navigators (HCNs) conducted follow-up telephone calls, targeting patients with high comorbidity.

Primary outcome was a newly updated advance directive (AD) within 90 days of the message. Secondary outcomes were a composite of clinically meaningful ACP (ACP billing codes, ACP notes, scanned ACP documentation [ADs, Physician Orders for Life-Sustaining Treatment forms]). Descriptive statistics were performed using available covariates. Analysis was conducted with SAS 9.4 (SAS Institute Inc); *P* < .05 indicated statistical significance.

## Results

A total of 2550 new surgical patients (1418 females [55.6%], 1129 males [44.3%]; mean [SD] age, 71 [9.0] years) received the EHR message, among whom 499 (24.3%) were also contacted by an HCN (Table 1). Compared with the message-only group, the message-plus-HCN group was more likely to have newly uploaded ADs (47 [9.4%] vs 74 [3.6%]; *P* < .001) and visit colorectal surgical clinics (130 [26.1%] vs 433 [21.1%]; *P* < .001). In 1 year, clinically meaningful ACP was documented in the EHR more often in the message-plus-HCN group vs message-only group (364 [72.9%] vs 311 [15.2%]; *P* < .001) (Table 2).

**Table 1.** Frequency of Newly Uploaded AD

Variable	No. (%)		*P* value
	Newly uploaded AD	No newly uploaded AD	
Age, mean (SD), y	71.5 (7.8)	71.0 (9.0)	.54
Gender identity			
Female	76 (62.8)	1342 (55.3)	.25
Male	45 (37.2)	1084 (44.6)	
Unknown	0	3 (0.1)	
Preferred language			
Chinese	0	71 (2.9)	.27
English	118 (97.5)	2170 (89.3)	
Spanish	0	60 (2.5)	
Other^a^	3 (2.5)	129 (5.3)	
Marital status			
Partnered^b^	73 (60.3)	1569 (64.6)	.61
Single^b^	29 (24.0)	551 (22.7)	
Unknown or declined to answer	3 (2.5)	71 (2.9)	
Widowed	16 (13.2)	239 (9.8)	
Charlson Comorbidity Index			
0-3	71 (58.7)	1228 (50.5)	.08
>4	50 (41.3)	1202 (49.5)	
Insurance type			
Commercial	17 (14.0)	332 (13.7)	.86
Medi-Cal	5 (4.1)	155 (6.4)	
Medicare	97 (80.2)	1900 (78.2)	
None	1 (0.8)	30 (1.2)	
Other^c^	1 (0.8)	13 (0.5)	
UCSF primary care	8 (6.5)	232 (9.5)	.28
UCSF palliative care	1 (0.8)	57 (2.3)	.27
Surgical clinic			
Colorectal	21 (17.4)	542 (22.3)	.19
Endocrine	27 (22.3)	504 (20.7)	
Hepatobiliary	46 (38.0)	1021 (42.0)	
Thoracic	27 (22.3)	362 (14.9)	

Abbreviations: AD, advance directive; UCSF, University of California San Francisco.

^a^ Other languages include Arabic, Burmese, Cambodian, Dari, Farsi, Gujarati, Hindi, Japanese, Khmer, Korean, Laotian, Mon-Khmer, Persian, Portuguese, Punjabi, Romanian, Russian, Tagalog, Thai, Tigrinya, Turkish, and Vietnamese.

^b^ Partnered is defined as being married, having a significant other, or having a registered domestic partner. Single is defined as single, divorced, or legally separated.

^c^ Other insurance include capitation plans.

**Table 2.** Frequency of Clinically Meaningful Advance Care Planning Between the Message Only Group and Message Plus Navigator Group

Outcome	No. (%)	
	Message only group (n = 2051)	Message + HCN group (n = 499)
Primary outcome		
Newly uploaded AD	74 (3.6)	47 (9.4)
Secondary outcomes		
New ACP *CPT* code	7 (0.3)	4 (0.8)
New ACP Notes	25 (1.2)	3 (0.6)
New ACP SmartLink	243 (11.8)	360 (72.1)
New scanned ACP documentation (AD, POLST forms)	90 (4.4)	51 (10.2)
Total new clinically meaningful ACP	311 (15.2)	364 (72.9)

Abbreviations: ACP, advance care planning; AD, advance directive; *CPT, Current Procedural Terminology*; HCN, health care nonclinical navigator; POLST, Physician Orders For Life-Sustaining Treatment.

## Discussion

Findings suggest that HCN support was associated with enhanced AD completion vs ACP messages alone. Routine messaging with HCN follow-up could increase ACP engagement without overburdening surgical practices.^3,4^

Study limitations include the short follow-up, which may not capture patients initiating but not completing an AD within 90 days. However, for this and other reasons (eg, patients engaging informally in ACP), the intervention likely had greater benefits than can be captured in the EHR. Additionally, given AD’s complex legal nature, both HCNs and patients encountered challenges in uploading documentation in the EHR. Efforts to simplify the legal execution and EHR upload for ADs may facilitate further availability in the EHR.

For older adults seeking consultation for major elective surgery, addition of HCN outreach to a standardized EHR message about ACP was associated with more ADs and ACP-related documentation. System-level resources are needed to simplify the documentation upload workflow.

## Notes

Supplement.
Data Sharing Statement
