# Supplementary material for: Health Care Navigators for Perioperative Advance Care Planning
Source: JAMA Netw Open. 2024 Jun 18;7(6):e2415452. doi: 10.1001/jamanetworkopen.2024.15452 (PMC11185963; doi:10.1001/jamanetworkopen.2024.15452)
Supplement: Supplement. — Data Sharing Statement [file jamanetwopen-e2415452-s001.pdf]

Data Sharing Statement

Colley. Health Care Navigators for Perioperative Advance Care Planning. *JAMA Netw Open*. Published June 18, 2024. doi:10.1001/jamanetworkopen.2024.15452

Data
Data available: No

Additional Information
Explanation for why data not available: Not able to de identify
